# Micro-TLC Approach for Fast Screening of Environmental Samples Derived from Surface and Sewage Waters

**DOI:** 10.1007/s10337-013-2445-3

**Published:** 2013-03-19

**Authors:** Paweł K. Zarzycki, Magdalena M. Ślączka, Elżbieta Włodarczyk, Michał J. Baran

**Affiliations:** Section of Toxicology and Bioanalytics, Koszalin University of Technology, Śniadeckich 2, 75-453 Koszalin, Poland

**Keywords:** Reversed-phase planar chromatography, Thermostated micro-TLC, Solid-phase extraction, Fluorescence, Phosphomolybdic acid, Environmental samples, Drinking water, Surface water, Sewage water, Endocrine modulators, EDCs, Multivariate statistics, Principal components analysis, Fingerprinting

## Abstract

In this work we demonstrated analytical capability of micro-planar (micro-TLC) technique comprising one and two-dimensional (2D) separation modes to generate fingerprints of environmental samples originated from sewage and ecosystems waters. We showed that elaborated separation and detection protocols are complementary to previously invented HPLC method based on temperature-dependent inclusion chromatography and UV-DAD detection. Presented 1D and 2D micro-TLC chromatograms of SPE (solid-phase extraction) extracts were optimized for fast and low-cost screening of water samples collected from lakes and rivers located in the area of Middle Pomerania in northern part of Poland. Moreover, we studied highly organic compounds loaded in the treated and untreated sewage waters obtained from municipal wastewater treatment plant “Jamno” near Koszalin City (Poland). Analyzed environmental samples contained number of substances characterized by polarity range from estetrol to progesterone as well as chlorophyll-related dyes previously isolated and pre-purified by simple SPE protocol involving C18 cartridges. Optimization of micro-TLC separation and quantification protocols of such samples were discussed from the practical point of view using simple separation efficiency criteria including total peaks number, log(product Δ*hR*_F_), signal intensity and peak asymmetry. Outcomes of the presented analytical approach, especially using detection involving direct fluorescence (UV366/Vis) and phosphomolybdic acid (PMA) visualization are compared with UV-DAD HPLC-generated data reported previously. Chemometric investigation based on principal components analysis revealed that SPE extracts separated by micro-TLC and detected under fluorescence and PMA visualization modes can be used for robust sample fingerprinting even after long-term storage of the extracts (up to 4 years) at subambient temperature (−20 °C). Such approach allows characterization of wide range of sample components that are present in given extract in high and middle concentration range. Due to protocol simplicity and low cost of analysis this method can be useful for preliminary sample screening.

## Introduction

Planar or thin-layer chromatography (TLC) is one of the distinctive separation tools that virtually do not require either a heavy equipment investment or extensive analytical staff training. In terms of fingerprinting and fast screening applications, particularly involving highly organic compounds loaded in the biological and environmental samples, modern planar separation systems based on high-performance thin-layer or ultra-thin monolithic plates (HPTLC, UTLC) are competitive to their column counterparts including high-performance liquid chromatography (HPLC) and capillary electrophoresis (CE) [1]. This is mainly because of parallel separation and sample processing and due to the fact that each analytical run can be performed on non-previously used stationary phase. Moreover, it is well recognized that planar chromatography may significantly reduce and simplify sample pre-purification and concentration protocol as well as offer number of detection and chromatogram acquisition possibilities [2, 3]. Low detection limits can be achieved via simple fluorescence, luminescence and biodetection or more sophisticated equipment based on, e.g. mass spectrometry including matrix-assisted laser desorption/ionization mass spectrometry (TLC–MALDI-MS), electron impact ionization mass spectrometry (TLC–EI-MS) or desorption electrospray ionization mass spectrometry (DESI-MS) [4–6]. Two-dimensional elution mode (2D) enables high-throughput separation of complex samples composed of more than 200 spots and such protocols were successfully applied for low-resolution metabolomic studies, using micro-TLC approach [1, 7–9]. In addition, miniaturized planar chromatography allows fast sample separation and low mobile phase consumption [10]. Therefore, mentioned technique can be recognized as green chemistry analytical tool. It should be noted that for majority of commercially available stationary phases including silica and octadecylsilane-bonded sorbents a migration distance within 50 mm allows satisfactory separation and high spot density that significantly improve detection of target substances [11–13]. Generally, modern high-performance planar chromatography including micro-TLC is an excellent illustration of how an old technique may have evolved in the past decade due to the development of both stationary phases as well as new detection and acquisition methods [1, 3, 11–16].

The presence of natural and anthropogenic-generated endocrine modulators in aquatic environment may cause potentially dangerous consequences to the humans and wildlife [17]. It has been found that numbers of such chemicals that mainly originated from pharmaceutical and food industry are finally discharged into environment through municipal treatment plants. Unfortunately, micro-pollutants considered as endocrine disrupting compounds (EDCs) cannot be only defined by their chemical nature but also mostly by their biological action, which strongly depends on concentration of target compounds and overall organic matrix composition [18]. The consequence of this is that many different classes of common pollutants can be collectively considered as EDCs and, therefore, simultaneous measurement of such substances in water ecosystems belongs to one of the challenging problem of modern analytical chemistry [19].

In this work we propose one and 2D micro-TLC separation protocols that are complementary to the previously elaborated HPLC method that was based on temperature-dependent inclusion chromatography and UV–Vis diode array detection [20]. Reported 1D and 2D micro-TLC protocols were optimized for fast and low-cost screening of water samples collected from lakes and rivers located in the area of Middle Pomerania in northern part of Poland as well as highly organic compounds loaded treated and untreated sewage waters, which were derived from municipal wastewater treatment plant “Jamno” near Koszalin City (Poland).

## Experimental

### Chemicals

7,8-Dimethoxyflavone (7,8-DMF) was product of Sigma (St. Louis, MO, USA). Methyl red (MR) was obtained from POCH SA (Gliwice, Poland). Phosphomolybdic acid (PMA) was purchased from Chempur (Piekary Śląskie, Poland). Methanol (LiChrosolv 99.8 % for liquid chromatography; Merck, Darmstadt, Germany) was used as received, without additional purification. Binary mobile phases were prepared using double-distilled tap water.

### Chromatography

Micro-TLC analysis was conducted on ready-to-use glass-based plates (100 × 100 mm) covered with RP18WF_254_S HPTLC stationary phase obtained from Merck. Before sample application, the factory-prepared plates were cut into small parts (50 × 50 mm) allowing 45 mm effective migration distance of the eluent front. Chromatographic process was performed inside temperature-controlled removable horizontal micro-TLC unit (chromium coated brass removable chamber) described previously [10]. Chromatographic chamber was working inside a foam-insulated metal oven connected to an external liquid circulating thermostat (Ultra-Low Refrigerated Circulator FP51-SL, Julabo, Seelbach, Germany) operating with ethanol as a circulating fluid. The system provided a constant and homogeneous temperature of TLC plate, which was set at 30 ± 0.02 °C. Chromatograms were generated under unsaturated chamber conditions using following running protocol: spotted with the samples micro-TLC plate was positioned horizontally inside a brass unit with the stationary-phase layer placed up side down. The unit was transferred into a temperature-controlled oven and sealed using a 1-mm thin glass cover. Afterwards, the movable cover of the oven was slid to reach the position above the TLC chamber unit and the temperature equilibration step was performed for 15 min. Chromatographic process was initiated after injecting a given eluent in a volume from 300 μL to 1 mL through an injection pipe into a mobile-phase application bar. TLC plate was removed from the chamber unit immediately after the mobile-phase front reached the plate edge located opposite to the application bar.

### Samples Application

One-dimensional chromatograms were spotted with 5 μL volume of each sample SPE extract. Particularly, 4-mm long bands were generated along start line (located 5 mm from the bottom edge of micro-TLC plate) using the spray-on technique involving Linomat 5 semi-automatic application instrument (Camag, Switzerland). This sampling machine was controlled through the Planar Chromatography Manager (winCATS software, 1999–2008, version 1.4.4.6337). In case of 2D chromatograms 1 μL of the sample extract was manually spotted in the right-bottom corner of the micro-plate (5 and 7 mm from the X and Y edges, respectively) using Hamilton-type glass syringe.

### Detection and Chromatograms Digitalization

Two modes of bands detection were applied including direct fluorescence and spot visualization after derivatization with PMA.

Fluorescence visualization was performed at *λ*_EX_ = 366 nm and *λ*_EM_ = Vis using a Cobrabid UV lamp (Warszawa, Poland). In such case TLC plate was placed on the black background, 8 cm from the light source and 10 cm from digital camera lens (the angle between lamp/plate/lens was 15°, approximately). Chromatographic pattern observed under visible light was acquired using an Olympus Camedia 5050 Zoom, 5.0 Mega pixel digital camera (Olympus Optical Co. Ltd., Japan) equipped with a 43-mm UV filter (Marumi, Japan). Digital shots were taken using the following camera settings: focusing mode—manual, lens mode—super macro, shutter speed 8 s, aperture F8.0, ISO sensitivity 64, recording mode RAW, image resolution 2,560 × 1,920. All Olympus RAW files (16 bits per RGB channel color deep mode) were transformed into an 8-bit TIFF file.

Spot detection was performed using visualization reagent that consisted of 10 % PMA in methanol. Developed micro-plates were dipped in glass jar filled with the PMA solution for 1 s, approximately. Blue-gray colored spots were generated after plates heating inside gravity convection oven (BMT Ecocell, Conbest; Krakow, Poland) for 20 min at 80 °C temperature. Picture acquisition after PMA visualization was performed using a Plustek OpticPro S12 USB scanner (Plustek, Taipei, Taiwan) with an 8-bit per RGB channel color deep mode, 600 DPI resolution and saved as TIFF files without compression with the help of image-acquisition software: Image Folio v. 4.2.0 (1991–2000, NewSoft Technology Corporation).

After data acquisition an appropriate TLC plate area was cropped from the original frame size. Quantitative data were obtained from unmodified files without additional image digital processing. Cross-sections of the chromatographic lanes and individual RGB pictures were extracted from the TIFF digital images using ImageJ software (ver. 1.42q Wayne Rasband, National Institutes of Health, USA; http://rsb.info.nih.gov/ij). In case of chromatograms included within figures presented in this paper, auto-balance conversion filter was applied to enhance the band contrast for satisfactory computer screen displaying and printing reproduction.

### Environmental Samples

In the current work we analyzed SPE extracts of surface and sewage water samples, which were previously generated for our study concerning determination of EDCs using temperature-dependent inclusion chromatography and involving HPLC system [20]. For this purpose the samples collection was conducted within 2007 and 2008 from lakes and rivers located in the area of Middle Pomerania in northern part of Poland. Treated and untreated sewage waters were collected from “Jamno” wastewater treatment plant near Koszalin City. After HPLC analysis the remaining volumes of collected SPE extracts were stored in the freezer at temperature of −20 °C until present micro-TLC separation. However, for optimization study of micro-TLC separation, sewage water extracts were prepared freshly using Supelclean LC-18 solid-phase extraction tubes (6 mL, 0.5 g) and involving detailed SPE analytical protocol for isolation and concentration of wide range of endocrine modulators from water samples [20].

### Data Analysis

Retention data were expressed as *hR*_F_ values derived from *R*_F_ data (*hR*_F_ = 100 *R*_F_). Evaluation of micro-TLC separation power involved Δ*hR*_F_ values calculated for the adjacent peaks. This parameter is commonly used as the optimization criterion to compare selectivity in the chosen TLC systems [12, 21]. Individual micro-TLC chromatographic profiles composed of multiple spots were compared using Δ*hR*_F_ product (ΠΔ*hR*_F_) similarly to optimization criteria proposed for column chromatography [22, 23]. In the present work logarithmic form of Δ*hR*_F_ product was applied due to wide (few factors) range of resulting ΠΔ*hR*_F_ values calculated for the spots visualized in studied samples.

Each collected sample (object) was characterized by 15 variables including selected physicochemical parameters (pH, water and air temperature and oxygen concentration) and clusters reflecting intensities of the chromatographic spots. Due to strong differences in the background matrix profiles and number of different peaks observed on the densitometric profiles recorded from individual environmental samples, each chromatogram was split and characterized by 10 clusters. Every specified cluster characterizes spots migrating within 0.1 *R*_F_ distance ranging from start line (*R*_F_ = 0) to eluent front (*R*_F_ = 1). Within each cluster individual peak groups were selected and their intensities (peak heights) were summarized. Such data were derived from fluorescence and PMA detection separately.

The experimental data were inspected with principal component multivariate statistical procedure using XLSTAT-Pro/3DPlot (version 2008.2.01) provided by Addinsoft, Paris, France.

## Results and Discussion

### Problem Overview

Effective fingerprinting of biological and environmental samples is one of the challenging analytical tasks. It involves complex matrix analysis for extremely wide range of target component concentrations. Planar chromatography due to specific principles including ability for parallel separation and sample processing as well as detection of all sample components on TLC chromatogram, including substances that are strongly adsorbed by the stationary phase (retarded or chemisorbed close to the start line), seems to be very interesting alternative for its column counterparts, especially HPLC and CE [1, 6, 11]. More importantly, the samples that are characterized by highly organic compound loaded matrices can be efficiently analyzed and quantified by planar chromatography including micro-TLC, where each separation can be performed using a new (non-previously-used) plate [12, 13].

Untreated sewage is an example of the most complex, unstable and extremely high organic substance loaded material [18, 19]. Usually, direct analysis of such sample is impossible or at least impracticable due to its instability. However, target substances from raw sewage can be relatively easily pre-purified and concentrated using classical solid-phase extraction (SPE) protocol, which may handle large volumes of liquid samples, e.g. classical 0.6 g tube is able to process 100–500 mL of raw sewage water. Previously, we optimized SPE protocol involving C18 cartridges and methanol/water binary phases for pre-purification, isolation and concentration of organic substances (mainly steroids) from various complex samples including blood, urine, surface water, treated and untreated sewage waters as well as activated sludge-based materials [20]. In this work we tested capability of micro-TLC for separation of low-molecular-mass substances from surface, treated and untreated sewage waters that can be detected under direct fluorescence and PMA visualization protocols. Such approach allows characterization of wide range of sample components that are present in given extract in high and middle concentration range. Due to protocol simplicity and low cost of analysis it can be useful for preliminary samples screening. Application of such separation and detection modes significantly extend fingerprinting capability that was demonstrated in our previous study involving HPLC and UV–Vis detection of EDCs (with polarity ranging from estetrol to progesterone) in surface water ecosystems [20].

### Method Development

**Fig. 1:**
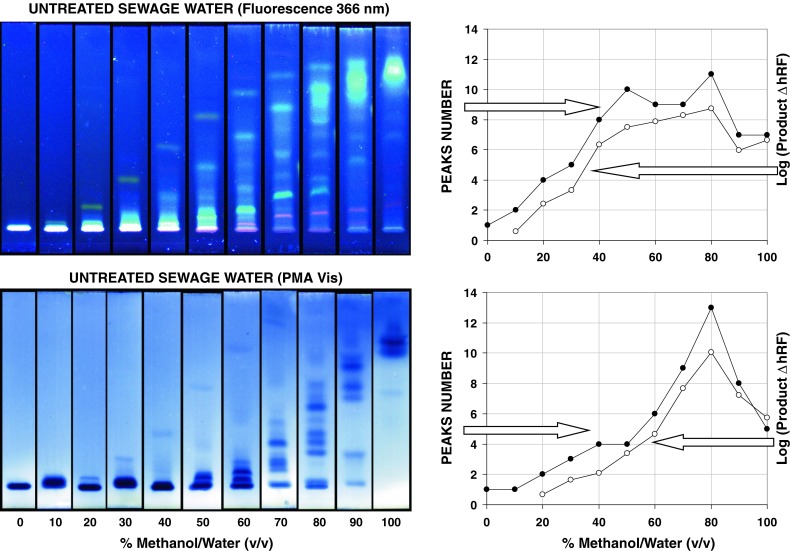
Separation and detection of SPE extracts of untreated sewage water using RP18WF_254_S HPTLC plates and different binary mobile-phase conditions (*left*) as well as corresponding selected separation criteria including peaks number and logarithmic form of Δ*hR*_F_ product (*right*)

**Fig. 2:**
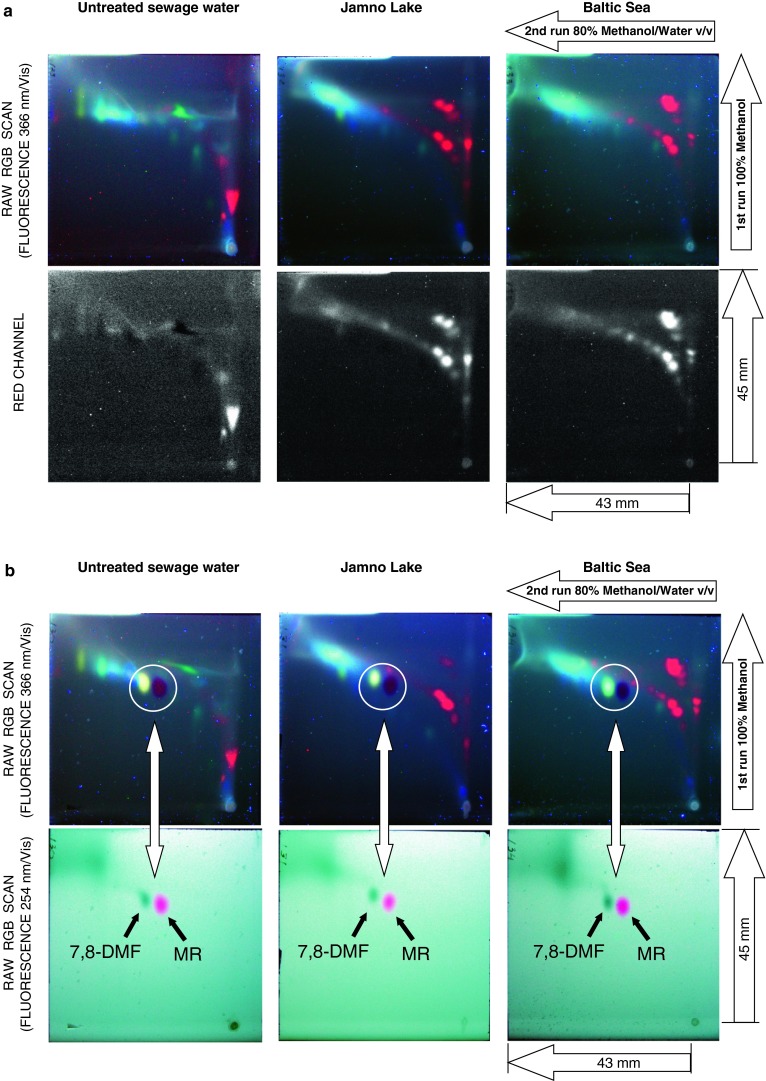
Two-dimensional chromatograms of environmental samples derived from sewage and different water ecosystems. **a** Raw samples (detection mode: fluorescence, *λ*_EX_ = 366 nm, *λ*_EM_ = Vis). **b** Samples spiked with retention markers: 7,8-dimethoxyflavone (7,8-DMF) and methyl red (MR); detection mode: fluorescence, *λ*_EX_ = 366 nm (*top*) and 254 nm (*bottom*), *λ*_EM_ = Vis

Typical SPE extract of untreated sewage water contains countless number of organic substances. However, for preliminary screening and sample fingerprints they can be relatively easily fractionated using reversed-phase separation system based on C18 water-tolerable plates and isocratic elution with methanol–water binary mobile phases (Fig. 1). Under such conditions separation of main components of the SPE extract start from 20 to 30 % of methanol for the peaks detected under fluorescence and PMA visualization conditions, respectively. For quantitative evaluation of separation power we used two simple criteria: total band (peaks) number and logarithmic form of Δ*hR*_F_ product calculated for the adjacent peaks. Considering the fact that front migration distance for each micro-plate was equal, these criteria can be directly applied for effective comparison of target component separation on different chromatograms. As can be seen from the optimization graphs presented within Fig. 1 the best separation can be achieved with mobile phase composed of 80 % (v/v) methanol in water. Under such conditions 12 and 13 individual bands were recorded on micro-plates, depending on the visualization mode selected. It should be noted that separation power of micro-plates can be significantly increased if 2D developing protocol is applied, which is well illustrated in Fig. 2a. Separation robustness of such complex samples can be evaluated by co-elution of retention markers, namely 7,8-DMF and MR. As can be seen from 2D chromatograms presented in Fig. 2b highly loaded organic compound matrix does not affect retention of individual compounds including proposed retention markers. Particularly, retention shifts do not exceed the peak base distance in both *X* and *Y* directions, measured for independent micro-TLC runs. We demonstrated that simple eluents composed of 100 % methanol (1st run) and 80 % of methanol in water (2nd run) allow effective separation of the number of red fluorescing bands that correspond to chlorophyll decomposition products. Such fingerprints are specific to given samples originating from untreated sewage water, Jamno Lake and Baltic Sea. Particularly, they may reflect the total algae activity in the surface water ecosystems investigated.

**Fig. 3:**
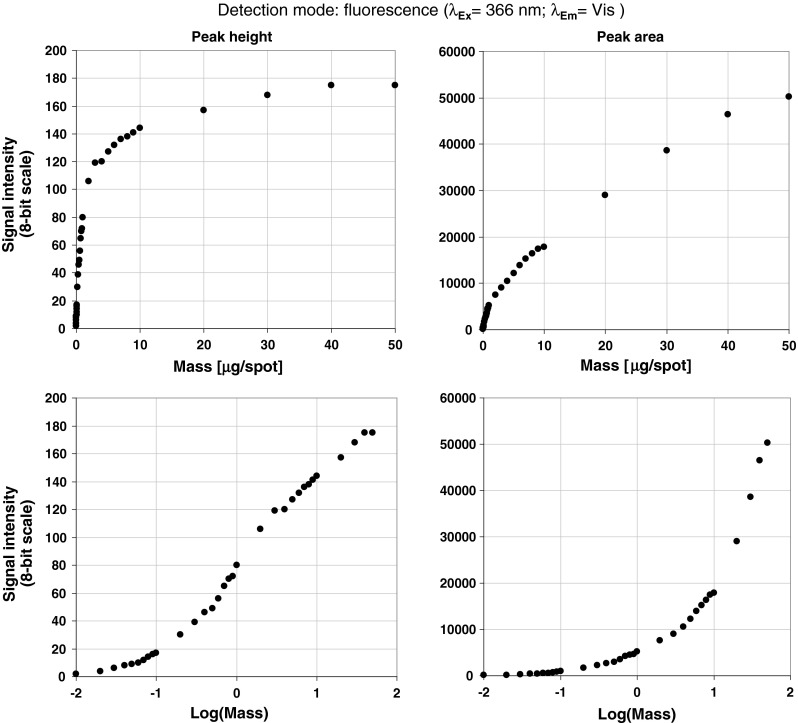
Detection response profiles for 7,8-dimethoxyflavone chromatographed on RP18WF_254_S HPTLC plates within mass/spot ranging from 10 to 50 μg. Signal intensity was derived from densitograms as the peak height (*left*) and peak area (*right*)

**Fig. 4:**
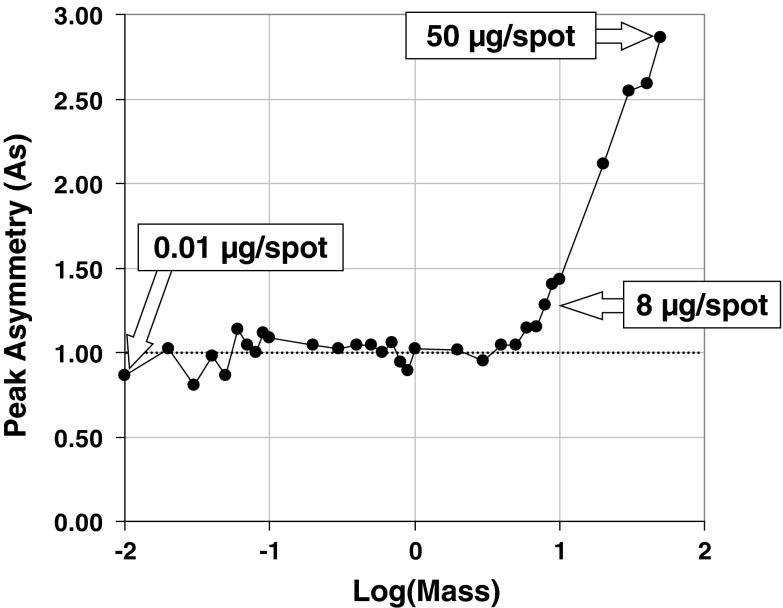
Changes in peak asymmetry (As) measured for different mass (from 10 to 50 μg) of 7,8-dimethoxyflavone

During analysis of surface and sewage waters samples, the analyst is usually dealing with unknown quantities of the substances extracted by SPE protocol. In this work we tested the capability of the image acquisition system working under fluorescence and visible light conditions using artificial marker: 7,8-DMF. This substance is migrating in the middle of RP18 W plate under 80 % of methanol in water conditions as well as giving a strong fluorescence within visible light area after exposure on UV light (366 nm). Particularly, we investigated the marker mass ranging from 10 ng to 50 μg spotted on TLC start line. The graphs concerning peak height and peak area signal responses (Fig. 3) have shown that the digital camera operating with the 8-bit acquisition mode is able to detect marker masses ranging over three factors. To evaluate capability of stationary phase to work with highly organic compound loaded samples we also measured the peak asymmetry within 7,8-DMF masses investigated (Fig. 4). This study has revealed the fact that symmetrical bands can be observed within wide range of mass investigated from 10 ng to 8 μg/spot. Micro-TLC process under RP conditions using the above-mentioned binary mobile phase was also evaluated by selected validation data concerning retention, band shape, mobile phase migration time within 45 mm developing distance and peak intensity (Table 1). In all cases the values of relative standard deviation (CV %) calculated for independent separation experiments are less than 10 %. This observation combined with 2D retention data presented for 7,8-DMF and MR markers strongly indicate that proposed separation protocol is satisfactory from reproducibility point of view and can be performed robustly using temperature-controlled micro-TLC process.

**Table 1:** Selected validation data concerning micro-TLC development and detection steps

	Retention factor^a^ (*R*_*F*_)	Peak asymmetry^a^ (As)	Plate developing time^a^ *t* (*s*)	Peak height^b^ (8-bit gray scale)
Average	0.47	1.02	905.32	137.25
Standard deviation	0.01	0.08	12.64	7.46
CV %	2.02	7.44	1.40	5.44

Analytical conditions: stationary phase—RP18W; mobile phase—80 % methanol:water; unsaturated and isothermic (30 °C) chamber; tested substance: 7,8-dimethoxyflavone

^a^Mass range: 0.01-7.0 μg/spot (*n* = 23)

^b^Mass 1 μg/spot (*n* = 13)

### Application of Micro-TLC Separation Protocol

**Fig. 5:**
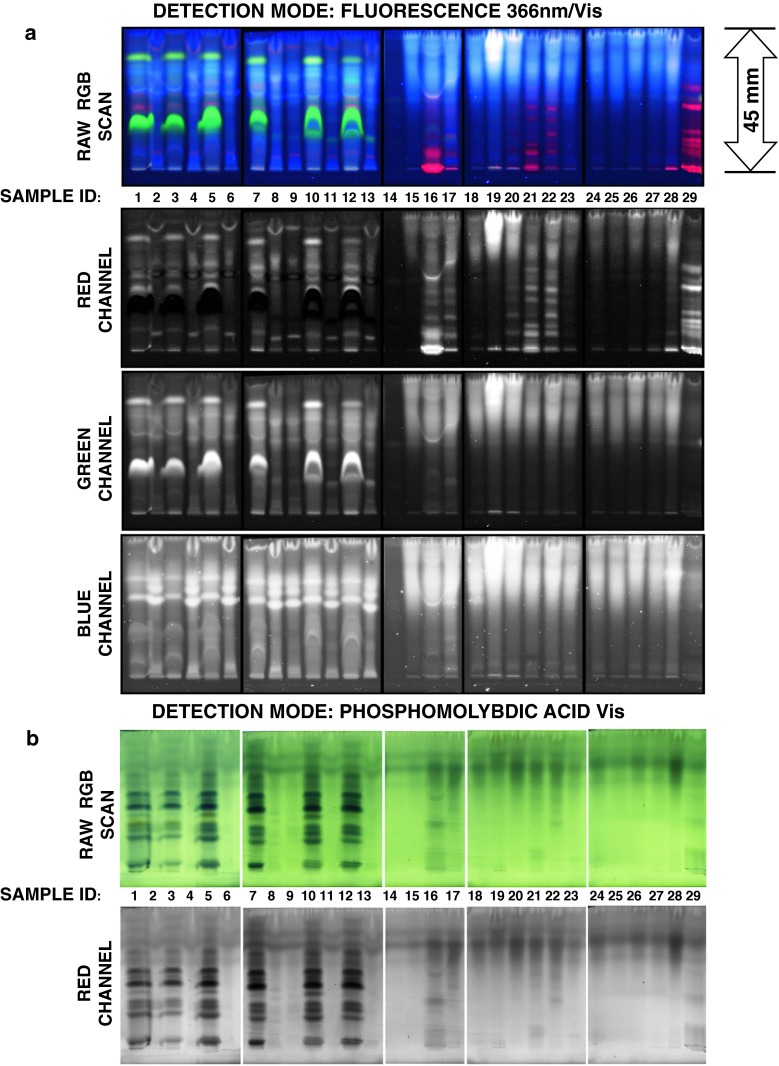
Micro-TLC chromatograms of SPE extracts derived from surface water ecosystems using HPTLC RP18WF_254_S plates, mobile phase consisted of 80 % methanol in water (v/v) and* fluorescence *(**a**) as well as PMA (**b**) detection. Samples I.D.: untreated sewage water: 1, 3, 5, 7, 10, 12; treated sewage water: 2, 4, 6, 8, 9, 11, 13; distilled water 14; tap water 15; Dzierżęcinka River (above Koszalin) 16; Dzierżęcinka River (under Koszalin) 17; Parsęta River 18; Radew River 19; Lubiatowo Lake 20; Jamno Lake 21; Bukowo Lake 22; Kamienne Lake 23; Kwiecko Lake 24; Stara Radew River 25; Rosnowo Lake 26; Hajka Lake 27; Parnowo Lake 28; Baltic Sea 29

For evaluation of micro-TLC method described above we separated the battery of SPE extracts of low-molecular-mass compounds derived from surface water ecosystems as well as treated and untreated sewage waters (Fig. 5). These samples were collected from 17 specific locations during our previous study involving HPLC technique based on temperature-dependent inclusion chromatography and direct UV–Vis detection of EDCs with polarity ranging from estetrol to progesterone [20]. It should be noted that sample collection was conducted between the years 2007 and 2008 and processed immediately using SPE technique. After that, obtained extracts were stored at temperature of −20 °C until HPLC quantification and present micro-TLC analysis. Surface water samples were collected from the lakes and rivers located in the area of Middle Pomerania in northern part of Poland while sewage waters were collected from “Jamno” wastewater treatment plant near Koszalin City (individual locations of 29 samples and their ID labels are listed within Fig. 5 caption). Generally, investigated water ecosystems are placed in the same geographical region; however, they are strongly differing in terms of limnological functions and they are characterized by different matrix of organic substances including endocrine-disrupting compounds, as we reported previously [20, 24]. Particularly, we investigated water samples from Baltic Sea, number of coastal and midland lakes including eutrophic, hypertrophic and mesotrophic areas as well as lowland rivers with moderate anthropogenic impact and receiving main stream of pollutants from local agriculture areas. Moreover, we also proceeded distilled and tap water from our laboratory as the low organic matrix reference samples.

Preliminary inspection of chromatographic lanes detected under fluorescence and PMA staining visualization conditions (Fig. 5) suggests that studied samples can be effectively fingerprinted and characterized using proposed micro-TLC protocol. Fluorescence acquired images contain number of red-fluorescing bands that correspond to chlorophyll dyes decomposition products and, therefore, may reflect total algae activity of water ecosystems investigated. On the other hand PMA visualization clearly discriminates highly organic compound loaded extracts, especially from untreated sewage water samples.

**Fig. 6:**
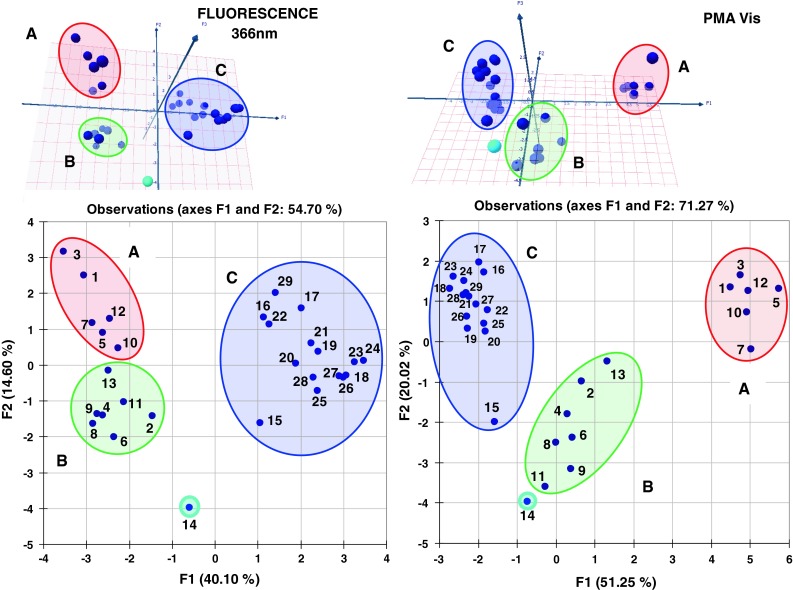
Principal component plots showing relationships between all objects investigated in respect to 1 and 2 factor scores (*bottom*) as well as 3D plot involving 1, 2 and 3 factor scores (*top*) based on fluorescence (*left*) and PMA (*right*) detection data sets. Individual object numbering corresponds to the sample ID, which are listed within Fig. 5 caption.* Clusters labels* untreated sewage water (**a**); treated sewage water (**b**); surface water (**c**)

**Fig. 7:**
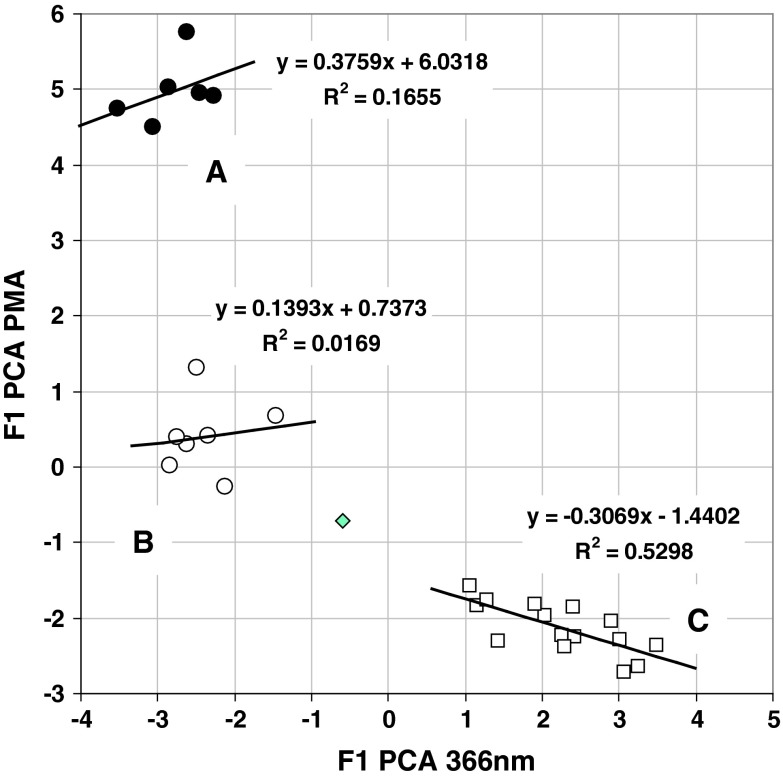
Relationship between F1 PCA factor scores calculated for fluorescence (*X*-axis) and PMA (*Y*-axis) detection data sets. **a**–**c** Objects groups are the same as specified in Fig. 6. *Diamond-shaped spot* corresponds to distilled water sample

Chemometric investigation based on principal component analysis (PCA) demonstrates that micro-TLC analysis can be used as complementary technique for environmental samples fingerprinting, similarly to HPLC/UV–Vis protocol described previously [20]. As can be seen from PCA graphs presented within Fig. 6 both fluorescence and PMA-derived data sets capture key information about origin of each sample. We may clearly recognize three clusters including spots corresponding to untreated (A), treated (B) and surface water ecosystems (C). Distilled water sample (ID = 14) is not grouping within extracts. This is evidently visible on both 2D and 3D graphs presented in Fig. 6. This indicates that observed clustering is predominantly caused by organic compound matrix isolated by SPE method. It is noteworthy to say that PCA sample clustering based on data derived from both detection methods may distinguish three individual objects groups; however, within each cluster the 1st factor score values are non-correlated (Fig. 7). This observation and the fact that there are differences in values of the total cumulative percent of the first and second principal components (F1 + F2) for the graphs presented in Fig. 6 strongly suggest that both visualization methods may capture complementary and discrete information concerning ecosystems investigated. This significantly extends fingerprinting capability of described methodology in comparison with chromatographic protocols based on column chromatography involving UV–Vis detection.

## Conclusions

Elaborated separation and detection modes of micro-TLC protocols significantly extend fingerprinting capability of our previous method involving HPLC and UV–Vis detection for profiling of endocrine-disrupting and related compounds.

Described micro-TLC analytical protocol allows characterization of wide range of sample components that are present in given extract in high and middle concentration range. Due to protocol simplicity and low cost of analysis it can be useful for preliminary samples screening.

Chemometric investigation based on PCA revealed that SPE extracts separated by micro-TLC and detected under fluorescence and PMA visualization modes can be used for robust samples fingerprinting of low-molecular-mass compounds, even after long-term storage of the extracts (up to 4 years) at subambient temperature (−20 °C).
